# The Effect of Multimodal Non-pharmacological Interventions on Cognitive Function Improvement for People With Dementia: A Systematic Review

**DOI:** 10.3389/fpubh.2022.894930

**Published:** 2022-07-12

**Authors:** Nigussie Tadesse Sharew

**Affiliations:** ^1^Department of Nursing, College of Health Science, Debre Berhan University, Debre Berhan, Ethiopia; ^2^Interdisciplinary Centre Psychopathology and Emotion Regulation (ICPE), University Medical Center Groningen, University of Groningen, Groningen, Netherlands

**Keywords:** dementia, cognitive function, multimodal intervention, old adults, systematic review

## Abstract

**Introduction:**

Dementia is a progressive brain degeneration characterized by a progressive deterioration in cognition and independent living capacity. Since dementia is a complex syndrome, multimodal non-pharmacological interventions (MNPIs) are highly recommended. Currently, there is less available evidence to describe the content, length, and frequency of multimodal interventions for cognitive function improvement for people with dementia (PWD).

**Method:**

A comprehensive search was performed in PubMed, EMBASE, CINAHL, Web of Science, and Medline international databases. The quality appraisal of the studies was done by the Cochrane risk of bias assessment tools.

**Results:**

A total of 19 controlled trial studies were included. Most of the included studies reported that MNPIs resulted in improvement, stability, or attenuation of decline in cognitive function of PWD. The reported effectiveness of MNPIs on cognitive function ranged from medium (0.29 Cohen's *d*) to large (2.02 Cohen's *d*) effect sizes. The median duration of intervention was 12 weeks for a 1-h session.

**Conclusion:**

This systematic review showed that MNPIs might improve people's cognitive functions for PWD. Physical exercise, music, and cognitive interventions were used in the content of multimodal interventions in a majority of the studies. Therefore, high-quality randomized controlled trial (RCT) studies with repeated-measured design on the combined effect of physical exercise, music, and cognitive intervention on cognitive function for PWD are recommended.

**Systematic Review Registration:**

http://www.crd.york.ac.uk/PROSPERO/, identifier: CRD42020222065.

## Introduction

Cognitive function decline is a major concern of old adults associated with an increased risk of mortality, disability, and poor quality of life (1, 2). According to the World Alzheimer's Disease International (ADI) report, dementia was one of the leading contributors to disability and dependency among the older population worldwide. Dementia is a progressive brain degeneration characterized by deterioration in cognition and independent living capacity (3). Every 3 s, at least one person develops dementia, and fifty million people live with dementia worldwide. This number will grow to 82 million by 2030 and 152 million by 2050 (4).

There is no treatment modality of pharmacological or non-pharmacological interventions that can cure dementia or halts its progression (5). Cholinesterase inhibitors are the most common pharmacological interventions with less evidence to support their efficacy (6). Believed to be safe with a few side effects, non-pharmacological interventions are the recommended types for dementia, including cognitive interventions, music therapy, reminiscence therapy, physical exercise, and many others (7).

Cognitive interventions are essential methods to improve cognitive function and prevent dementia. Cognitive interventions are mainly divided into three main categories, i.e., cognitive rehabilitation, cognitive stimulation, and cognitive training (8). First, cognitive rehabilitation interventions are emphasized on improving and maintaining cognitive function related to daily activities by enhancing independent living (9). These types of interventions are most effective for chronic and more progressive patients. Second, cognitive stimulation interventions, including discussion, reality orientation, and reminiscence therapy, had positive impact on cognition in patients with mild to moderate dementia (10). Finally, cognitive training is a non-pharmacological intervention involving regular mental activities that help maintain or even increase a person's cognitive abilities. These interventions effectively improved general cognitive functioning for people with mild to moderate dementia (11).

Non-pharmacological interventions can be delivered separately or in the format of a multimodal approach. Multimodal non-pharmacological interventions (MNPIs) combine two or more types of non-pharmacological interventions (12). Multimodal approaches are currently recommended for treating people with dementia (PWD) (13, 14).

There are different studies on the effect of MNPIs on cognitive function in PWD. For example, a meta-analysis study conducted in the USA found small improvements in selected cognitive abilities in early-stage dementia from a combination of cognitive training and physical exercise (15). In addition, the study combined music and physical exercise intervention showed that physical exercises matched to rhythmical and enjoyable music appear to improve the cognitive function of PWD (16). Furthermore, the study conducted in Germen showed that 6-month MNPIs that combine motor stimulation, daily living, and cognitive stimulation improved cognitive function among PWD in nursing homes (17).

A systematic review was performed to evaluate the effects of MNPIs on cognitive function among PWD living in a nursing home (7). This review included studies conducted on patients in nursing homes that have their unique standards and resources affecting cognitive function improvement for the clinical population. Therefore, the result of this systematic review might not be generalized to both PWD living in the community and residential care facilities. Thus, the current systematic review was conducted to investigate the available evidence on the content, frequency, and length of intervention of MNPIs in cognitive function for PWD living in residential care facilities and the community. The current review will also provide a basis for establishing guidelines and recommendations for future multimodal intervention for old adults with dementia.

## Study Design and Search Strategy

The protocol of this systematic review was registered in the International Prospective Register of Systematic Reviews (PROSPERO) with the registration number CRD42020222065. In addition, the preferred reporting items for systematic review and meta-analysis (PRISMA) statement (18) were utilized to write this systematic review.

Multimodal non-pharmacological interventions have been indicated in different studies such as multimodal, integrated, multicomponent, multidomain, mixed, and combined. Therefore, the current author used these search terms for MNPIs to include multimodal intervention comprehensively. Searches were conducted in PubMed, EMBASE, CINAHL, Web of Science, and Medline international databases from inception to 31 December 2020. The author also used a variety of key search terms combining Medical Subject Headings (MeSH) and free-text terms of “Dementia,” “Alzheimer's Disease,” “Combine,” “Multimodal,” “Old adults,” “Elderly,” “Older residents,” “Cognitive function,” and “Randomized Controlled Trial” (Supplementary Table 3: Search strings in each database). Missed data were handled by contacting the corresponding author/s. The EndNote (version X9) reference management software for windows was used to download, organize, review, and cite the articles.

## Eligibility Criteria

Studies were considered eligible for screening if they used PWD as population; MNPIs as intervention; unimodal non-pharmacological interventions or control group with no intervention as comparison group, and cognitive function as the outcome. All studies included in this review met the following inclusion criteria: (1) controlled trial studies, (2) involved older people with a primary diagnosis of dementia, (3) had at least two modes of non-pharmacological intervention, (4) assessed cognitive function as an outcome measure, and (5) studies published in the English language. Studies were excluded if they had pharmacological intervention as one content of the intervention and were published in a language other than English, abstracts without full text, pilot studies, and symposium/conference proceedings and essays.

## Selection Process

Nigussie Tadesse (NT) and Tesfa Dejenie (TD) screened articles independently from the title and abstract based on the inclusion and exclusion criteria after conducting the deduplication process. Disagreements on including titles and abstracts were solved through discussion and consensus at the zoom meeting. After removing irrelevant studies, full articles were screened for final review by NT and TD. Consensuses of the two reviewers resolved any discrepancies.

## Data Extraction and Data Synthesis

The Cochrane collaboration data collection format for randomized and non-randomized control trial studies was utilized to extract data. The data extraction was done with the first author, year of publication, number of included studies and participants, participants allocation, duration and session of interventions, the content of intervention for each group, description of activities in each session, setting of intervention, and provider of intervention and outcome measures. Consensuses of the two reviewers resolved any discrepancies in data extraction. The author also used various data formats, including mean difference, standard means difference, standard error, F-statistics, and sample size, to calculate effect size in outcome measure if not reported in the studies (19). In addition, the author has evaluated the effect sizes according to Cohen's criteria which stated that 0.2, 0.5, and 0.8 represented small, medium, and large effects, respectively (20). However, a meta-analysis was not performed since there was heterogeneity in intervention content for control and intervention groups in each study.

## Quality Appraisal

Quality appraisal of the peer-reviewed literature utilized the risk of bias in the non-randomized control trial studies I (ROBINS-I) tool for non-randomized control trial studies (21) and the Cochrane Risk-of-Bias tool for randomized trials (RoB 2) revised 2019 (22). The author and Tesfa Dejenie independently evaluated each included study's quality, and consensus was reached by discussing discrepancies in the quality appraisal.

The ROBINS-I tool's overall quality appraisal judgment was either low, moderate, or serious and critical risk. The study is judged to be at a low risk of bias in which all domains had low risk. If the study had at least moderate risk but no critical risk and serious risk in one domain, it was judged to be a moderate risk. If the study had at least one serious risk of bias in one of the domains but no critical risk of bias, it was judged to be a serious risk. Finally, if the study had at least one critical risk in one domain, it was judged to be a critical risk (21).

In the RoB tool for randomized trials tool, the overall quality appraisal judgment was done either of low risk, some concern, or high risk. First, the study is judged to be at low risk of bias in which all domains had low risk. Second, the study is judged to raise some concern in at least one domain of the result that had some concern but not at high risk of bias. Finally, if the study had at least one high risk of bias in one of the domains or more than one “some concern,” it was judged to be a high risk of bias (22).

## Results

A total of 998 studies were extracted through the database search. After screening all records, 74 full-text articles were assessed for eligibility. Finally, 19 studies were included in the systematic review. Physical exercise was classified into aerobic, strength, and stretching exercises and considered these exercises as one intervention content (7). In this systematic review (23), using a three-arm intervention to compare the effect of the combined aerobic and strength exercise with a social visit and aerobic exercise group by considering these exercises had different effects. In addition, one of the included studies was conducted among healthy adults, people with mild cognitive impairment (MCI), and PWD, in which the author looked at the effectiveness of computerized cognitive training and reminiscence therapy among PWD (24). Supplementary Figure S1 shows the PRISMA flow diagram of the study selection process.

This systematic review included 14 RCTs (23–36) and 5 quasi-experimental studies (37–41).

## Quality Appraisal Results

Of the 14 RCT studies, seven had an overall low risk of bias, five studies had some concern risk of bias, and two articles had a high risk of bias (33, 34) (Supplementary Figure S2). Of the five non-RCT studies, three were assessed as having moderate risk and two as low risk (Supplementary Figure S3).

## Study Area and Participants

Studies were from Brazil (4), Hong Kong (3), the Republic of Korea (3), The Netherlands (3), Japan (1), German (1), Greek (1), Taiwan (1), and Portugal (1) and one study was from Italy, Greece, Norway, and Spain. The number of participants in the systematic review study ranged from 17 (28) to 165 (26) with a mean of 59, and the total sample size in this systematic review was 1,123. Among the study participants, 68.57% (770) were women, and all participants were older than 65 years. Most of the studies recruited participants from residential care facilities, and a two-arm controlled trial was employed. Supplementary Table S1 shows the study characteristics of included studies.

## Contents of Non-Pharmacological Interventions

The contents of non-pharmacological intervention used in the multimodal interventions were as follows: 18 exercises, 11 cognitive pieces of training, eight music therapies, four cognitive stimulations, two art, two horticulture, one reminiscence, one activity of daily living (ADL), one cognitive rehabilitation, one spiritual element, one recreation activity, one handicraft, one dyadic-based multicomponent intervention, and one occupational therapy. Regarding the number used in MNPIs, four articles (27, 31, 34, 39) used six non-pharmacological interventions.

## Frequency, Duration, and Length of a Multimodal Intervention

The duration of interventions ranges from 6 weeks (26) to 48 weeks (27). The multimodal intervention had a range of one session per week (26, 28, 33) to 15 times a week (31); the length of session ranged from 30 min (26) to 300 min per session (34); and the total number of hours of intervention ranged from 6 h (26) to 576 h (27). The median duration of intervention was 12 weeks with 1 h of the session, and most of the studies were intervened for more than three sessions per week.

## Outcome Measure

Different cognitive assessment tools were utilized to evaluate the effectiveness of the interventions in which 15 studies measured global cognition function, and others (25, 30, 32, 37) used specific cognitive function. In this review, 12 studies utilized Mini-Mental State Examination (MMSE), two studies used Alzheimer's Disease Assessment Score—Cognitive subscale (ADAS—Cog), and other studies used Montreal Cognitive Assessment (MoCA), Trail Marking Test—Part A (TMT—A), Clock Drawing Test (CDT), Frontal Assessment Battery (FAB), and other types of tools to measure specific cognitive functions.

## Effectiveness of MNPIs

The majority of the included studies [except (28, 33, 34)] reported that MNPIs effectively improved or stabilized cognitive function in PWD with medium (Cohen's *d* 0.29) to large (Cohen's *d* 2.02) effect sizes. Four of the included studies resulted in larger effect sizes (Cohen's *d* > 0.8) (29, 38, 40, 41). These studies were conducted on people with mild to moderate dementia. Supplementary Table S2 shows intervention descriptions and outcome measures of the included studies.

## Discussion and Clinical Implication

In this systematic review, most of the included studies (16/19) reported that MNPIs resulted in improvement, stability, or attenuation of decline in cognitive function of PWD. The MNPIs identifying individuals' needs of patients and tailoring interventions resulted in the most significant improvement in cognitive function among PWD. This result was also supported by a study conducted in nursing homes (7).

Fifteen studies were conducted in residential care units, and four studies (30, 32, 37, 38) were community-based interventions. As the number of people living with dementia rises, more people tend to live in their homes to maintain their independence. Therefore, community-based interventions are relevant to involve family members in the intervention, like dyadic-based interventions that enhance the implementation of MNPIs. This study finding was supported by another systematic review that reported that community-based interventions effectively improved cognitive function, quality of life, and ADL (42). Therefore, in addition to residential-based intervention, community-based interventions also effectively improved the cognitive function of PWD.

In this systematic review, the MNPI had a range of 1–6 sessions per week and 6 weeks to 48 weeks of a duration of intervention. Eight studies employed at least three sessions per week, and 13/19 were conducted for 12–48 weeks. In addition, most of the interventions were provided in an integrated approach in groups. Furthermore, since dementia is a multifactorial disease, an integrated intervention increases patients' perceptual orientation by motivating them to use cognitive, physical, emotional, and spiritual abilities. Therefore, integrated group-based MNPIs were effective in helping patients to stabilize their emotional health and share personal experiences resulting in cognitive improvement.

In this systematic review, 11 studies used cognitive training such as memory activities like generating words according to semantic criteria (naming of animals, fruits, and flowers) and memory games such as chopsticks hand game, simple chess, puzzling, count backward, logical reasoning, language, orientation, simple calculation, and drawing with pen and pencil. These cognitive training activities stimulate learning and memory, reality orientation, and multisensory stimulation. This study finding was supported by the study conducted in the USA that memory activities resulted in social and mental stimulation for those with dementia while “exercising” the brain and, possibly, slowing cognitive abilities' deterioration (43).

The study conducted in the Netherlands showed that multicomponent dyadic intervention, which included physical exercise and supported the existing psycho-education, communication skills training, and pleasant activities training, was effective in maintaining function for PWD (32). In this study, participants were engaged in flexibilities, balance, strengthening, and endurance with a coach in their home. This study was also supported by a survey conducted in the USA in which dyadic-based multicomponent intervention resulted in improved cognitive function (44). The dyadic intervention was relevant to enhanced social chatting and increased adherence to any intervention. PWD usually feels depressed and lonely, which contributes to a decline in cognitive function. Therefore, dyadic-based interventions having multicomponent interventions are highly recommended.

In this systematic review, 94.7% of the studies utilized physical exercises as one content of the multimodal intervention. Physical exercises, like Tai chi exercise, have improved cognitive function in the early stage of dementia, as supported by a systematic review (45). In addition, a study conducted on the effect of physical exercises in people with cognitive decline resulted in reduced muscle weakness, stress, and depression for PWD, which is used to boost memory and thinking abilities (46). Some studies also indicated that higher fitness levels and a longer duration of physical exercise had a positive effect on the volume of the hippocampus, which mediates improvement in memory (47). Therefore, combining physical exercise with other non-pharmacological interventions might increase the probability of cognitive function improvement.

Multimodal intervention combining Tai chi exercise and cognitive stimulation therapy resulted in moderate to large effect size improvement on cognitive function (35, 36). Tai chi is a type of exercise that includes cognitive, social, and mediation components. This exercise involves sequential pattern movement, which requires visuospatial skill, rapid information processing, and episodic memory (48). Cognitive stimulation therapy also optimized cognitive function for PWD within a socially oriented context through an integrated approach (49). PWD learns to coordinate activities and refreshes with social chatting while performing Tai chi and cognitive stimulation therapy in the group. Therefore, combining Tai chi exercise and cognitive stimulation therapy might enormously enhance the cognitive function of PWD.

Four studies in this systematic review were conducted by combining music and physical exercise. Combining music with exercise improved cognitive function (26). In this study, music-with-movement using props, such as a balloon, ribbon, balls, rhythmical tapping of the feet, and mirroring movement was intervened. In addition, a study conducted in Portugal on the combined effect of multicomponent exercise and music resulted in improved cognitive function in PWD (41). This study finding was supported by a study conducted in Greek combining aerobic, music therapy, and memory game (29). Another study combined singing their familiar music with exercise like walking while singing or playing simple musical instruments improved attention (25). Finally, the effectiveness of combining physical exercise and music was supported by another review, showing that longer durations of physical exercises matched to rhythmical and enjoyable music appear to improve cognitive function (16). Therefore, integrating musical therapy with physical exercise for PWD might synergistically enhance cognitive function.

In this systematic review, all studies combined physical exercise, music, and cognitive intervention with each other and other NPIs. These three modes of intervention improved cognitive function differently. First, music therapy positively impacted cognitive function's memory and verbal fluency component (50). Second, cognitive intervention improved brain function of attention, cognitive flexibility, problem-solving, reasoning, and working memory (51). Last, physical exercise improved memory performance by gaining cardiorespiratory fitness (52) and increasing neuroplasticity in the hippocampus. Therefore, combining physical exercise, music, and cognitive interventions might have a synergetic effect in improving cognitive function or delaying cognitive function decline for PWD.

## Limitations and Strengths of the Study

This systematic review has some limitations and strengths. Some of the limitations include even though comprehensive search terms were utilized to explore studies, specific cognitive functions, such as visuospatial function and executive function outcome of interventions for dementia, were not sufficiently extracted. In addition, the wide diversity of nomenclatures of multimodal interventions and the availability of a variety of combinations of non-pharmacological interventions might affect the comprehensiveness of the search strategies and miss some studies. Furthermore, articles published other than English were excluded, which might affect the external validity. However, besides the above limitations, this study has some noteworthy strengths. All of the included studies were more recent controlled trial studies. Therefore, the evidence of this systematic review will be strong with high internal validity for policymakers, health professionals, and PWD. In addition, updated quality appraisal Cochrane tools were utilized to evaluate the quality of included studies.

## Conclusion

This systematic review of the control trial studies showed that MNPIs might improve people's cognitive function. The effectiveness of MNPIs for the cognitive function ranged from medium to large effect sizes, which vary according to the degree of cognitive decline. Almost all the implemented MNPIs include exercise as one integral part of their intervention. The majority of the studies were conducted for more than 12 weeks and three sessions per week. Most of the included studies combined physical exercise, music, and cognitive intervention in the content of their multimodal interventions. High-quality RCTs with repeated-measured design on the combined effect of physical exercise, music, and cognitive intervention on cognitive function for PWD are recommended. Moreover, this review included few community-based multimodal interventions, and further research in this area may be intended. Furthermore, the included studies were largely heterogeneous in their content of the intervention, duration, and outcome measurement scales. Therefore, further work is needed from specific to multifactorial studies in this area.

## Data Availability Statement

The original contributions presented in the study are included in the article/Supplementary Materials, further inquiries can be directed to the corresponding author/s.

## Author Contributions

NT was responsible for drafting and finalizing this systematic review in collaboration with other senior professionals from the Hong Kong Polytechnic University and Maastricht University.

## Conflict of Interest

The author declares that the research was conducted in the absence of any commercial or financial relationships that could be construed as a potential conflict of interest.

## Publisher's Note

All claims expressed in this article are solely those of the authors and do not necessarily represent those of their affiliated organizations, or those of the publisher, the editors and the reviewers. Any product that may be evaluated in this article, or claim that may be made by its manufacturer, is not guaranteed or endorsed by the publisher.
