# Supplementary material for: The Effect of Multimodal Non-pharmacological Interventions on Cognitive Function Improvement for People With Dementia: A Systematic Review
Source: Front Public Health. 2022 Jul 12;10:894930. doi: 10.3389/fpubh.2022.894930 (PMC9314571; doi:10.3389/fpubh.2022.894930)
Supplement: Supplementary file 1 [file Table_1.pdf]

Supplementary Table 1: Study characteristics of included studies, 2021

Autor (Year)	Country	Sample size	Sex (M/F)	Mean age	Study design	Study Setting	Intervention Provider
(Barban et al., 2016)	Italy, Greece, Norway, and Spain	EG=42<br>CG=39	EG: 13/29<br>CG: 11/28	EG: 76.7 (5.7)<br>CG: 76.9 (5.7)	Crossover, single-blind, two-arm RCT	Residential care unit	Cognitive therapist
(Bossers et al., 2015)	The Netherlands	EG=37<br>CG_1=36<br>CG_2=36	EG=8/29<br>CG_1= 8/28<br>CG_2=11/25	EG= 85.7 (5.1)<br>CG_1=85.4 (5.4)<br>CG_2=85.4 (5.0)	Parallel, single-blind three-arm RCT	Residential care unit	Trained professional
(Chen & Pei, 2018)	Taiwan	EG=15<br>CG=13	EG: 6/9<br>CG: 8/5	EG: 77.3 ± 9.4<br>CG: 77.3 ± 10.0	Parallel, Open-labelled two-arm RCT	Residential care unit	Music therapist
(Cheung et al., 2018)	Hong Kong	EG=58<br>CG_1=54<br>CG_2=53	EG=15/43<br>CG1= 12/42<br>CG2=13/40	EG= 85.71 (6.68)<br>CG_1= 84.50 (6.82)<br>CG_2= 85.58 (7.46)	Parallel, single-blind, three-arm RCT	Residential care unit	Investigator
(Coelho et al., 2013)	Brazil	EG=14<br>CG=13	NR	EG=78.0±7.3<br>CG=77.1±7.4	Parallel, quasi-experimental with two arms	Community	NR

(de Andrade et al., 2013)	Brazil	EG=14<br>CG=16	EG: 2/12<br>CG: 4/12	EG=78.6±7.1<br>CG=77.0±6.3	Parallel, quasi-experimental with two arms	Community	NR
(Graessel et al., 2011)	German	EG=50<br>CG=46	EG: 6/44<br>CG: 10/36	EG: 84.5 (4.5)<br>CG: 85.7 (5.7)	Parallel, single-blind, two-arm, longitudinal RCT	Residential care unit	Trained nurse
(Higuti et al., 2020)	Brazil	EG=9<br>CG=8	NR	EG= 79.6(9.3)<br>CG=79.0(12.6)	Parallel, double-blind, two-arm, pilot RCT	Residential care unit	Trained professional
(Kampragkou et al., 2017)	Greek	EG=15<br>CG=15	NR	NR	Parallel, double-blind, two arm RCT	Residential care unit	Trained therapist
(Kang et al., 2010)	Republic of Korea	EG=20<br>CG=18	EG: 4/16<br>CG: 0/18	EG=Majority 65-79<br>CG= Majority 65-79	Parallel, quasi-experimental with two arms	Residential care unit	Trained professional
(Karssemeijer et al., 2019)	The Netherlands	EG=38<br>CG_1=39<br>CG_2=38	EG=20/18<br>CG1= 21/18<br>CG2=21/17	EG= 79.0 (6.9)<br>CG_1= 80.9 (6.1)<br>CG_2=79.8 (6.5)	Parallel, single-blind, three-arm RCT	Community	Trained professional
(Kim et al., 2016)	Republic Korea	EG=19<br>CG=14	EG: 6/13<br>CG: 2/12	EG=81.9(7.0)<br>CG=80.9(6.1)	Parallel, double-blind, two-arm RCT	Residential care unit	Physical therapist

(Lee et al., 2015)	Republic of Korea	EG=13<br><br>CG=13	EG: 3/10<br><br>CG: 2/11	EG=80.15(5.21)<br><br>CG=80.00(6.90)	Parallel, quasi-experimental with two arms	Residential care unit	NR
(Prick et al., 2017)	The Netherlands	EG=57<br><br>CG=54	EG: 31/26<br><br>CG: 39/15	EG=76(7.61)<br><br>CG=78(7.17)	Parallel, open-label, two-arm RCT	Community	Trained professional
(Sampaio et al., 2019)	Portugal	EG=19<br><br>CG=18	EG: 4/15<br><br>CG: 5/13	EG= 84.8(5.9)<br><br>CG= 83.3(5.3)	Parallel, quasi-experimental with two arms	Residential care unit	Trained professional
(Satoh et al., 2017)	Japan	EG=31<br><br>CG=31	EG: 2/29<br><br>CG: 2/29	EG=87.0(5,4)<br><br>CG=87.4(4.4)	Parallel, open-labelled, two-arm RCT	Residential care unit	Trained social worker
(Viola et al., 2011)	Brazil	EG=20<br><br>CG=16	EG: 7/13<br><br>CG: 6/10	EG=Aver. 75<br><br>CG=Aver. 75	Crossover, single blind, two-arm, RCT	Residential care unit	Trained professional
(Young, 2020)	Hong Kong	EG=41<br><br>CG=39	EG: 7/34<br><br>CG: 8/31	EG: 80.05 (6.17)<br><br>CG: 80.25 (6.33)	Parallel, double-blind, two-arm RCT	Residential care unit	Trained social worker
(Young et al., 2019)	Hong Kong	EG=51<br><br>CG=50	EG: 10/41<br><br>CG: 10/40	EG: 80.53 (6.26)<br><br>CG: 79.86 (6.59)	Parallel, double-blind, two-arm RCT	Residential care unit	Trained social worker

Abbreviations: NR: Not recorded, EG: Exposed group, CG_1: Control group 1, CG_2: Control group 2, RCT: Randomized controlled trial
