# Supplementary material for: The Effect of Multimodal Non-pharmacological Interventions on Cognitive Function Improvement for People With Dementia: A Systematic Review
Source: Front Public Health. 2022 Jul 12;10:894930. doi: 10.3389/fpubh.2022.894930 (PMC9314571; doi:10.3389/fpubh.2022.894930)
Supplement: Supplementary file 2 [file Table_2.pdf]

Supplementary Table 2: Intervention descriptions and outcome measures of the included studies, 2021

Authors, year	Intervention description			Outcome measuring tools and evaluation results
	Content of interventions	Description of activity per session	Frequency and duration of interventions	
(Barban et al., 2016)	EG = Computerized cognitive training and Reminiscence Therapy (RT)<br><br>CG = Rest for the first round	Activities / session for EG<br><br>-1 hour/session:<br><br>Multicomponent process-based cognitive training:<br><br>1) 30 minutes which includes 10 minutes for memory, 10 minutes for executive function and 10 minutes for other cognitive exercise like logical thinking and language<br><br>2) RT for 30 minutes by asking question about autobiographical data<br><br>Activities / session for EG<br><br>Crossover RCT was conducted and the participants in the	For EG<br><br>- 2 session/week for 12 weeks	Outcome measurement tool: Mini Mental State Examination (MMSE)<br><br>In this study there were three arms based on the diagnosis of the participants i.e., healthy ageing, Mild Cognitive Impairment (MCI) and Mild Alzheimer's Diseases (mAD) and for this study we only considered mAD groups only<br><br>Outcome evaluation: Computerized cognitive training and reminiscence therapy resulted in significant improvement of in MMSE for mAD participants (F (2,158) = 6.085, P = 0.004); ES = 0.55 Cohen's *d*.

		controlled group were at rest.		
(Bossers et al., 2015)	EG= Anaerobic and Strength exercise<br><br>CG_1 = Anaerobic exercise only<br><br>CG_2 = Social visit	<u>Activities / sessions for EG</u><br><br>one hour/ session:<br><br>- Strength exercise focused on lower-limb strengthening and high-intensity walking exercise for 30 min<br><br><u>Activities / session for CG_1</u><br><br>- Walking exercise for 30 minutes<br><br><u>Activities / session for CG_2</u><br><br>-Social visits for 30 minutes	<u>For each group</u><br><br>- 4 sessions/week for 9 weeks	Outcome measurement tool: MMSE<br><br>Outcome evaluation: Combination of aerobic and strength exercise training is more effective than social group in improving global cognitive function with mean difference of 0.43; 95% confidence interval 0.176- 0.685 t (71) = 4.12; ES = 0.43 Cohen's *d*.
(Chen & Pei, 2018)	EG = Musical Dual - Task Training (MDTT)<br><br>CG = Non-musical cognitive task and cognitive exercise	<u>Activities / session for EG</u><br><br>-one hour/session<br><br>Singing and walking or stepping<br><br><u>Activities / session for CG</u><br><br>-one hour playing card, chess, reading and walking	<u>For each group</u><br><br>-1 session/week for 8 weeks	Outcome measure tool: Trail Marking Test – Part A (TMT-part – A) was used to measure executive function.<br><br>Outcome evaluation: Musical dual -task program resulted in attention control function improvement in decreasing TMT-part A score significantly in MDTT group (t (1,14) = 6.6, *P* = 0.01), but not in control group and cumulative effect size was not reported.

(Cheung et al., 2018)	EG = Music-with-movement (MM) intervention<br><br>CG_1 = Music Listening (ML)<br><br>CG_2 = Social activities	Activities /sessions for EG<br><br>-30 minutes:<br><br>-5 min greeting exercise<br><br>-20 min MM<br><br>Activities /sessions for EG<br><br>-5 min closing exercise<br><br>Movement to music using props, such as balloons, ribbons, balls, rhythmical tapping of the feet and mirroring movement shown by a teacher<br><br>Activities /session for CG_1<br><br>Listening preferred music<br><br>Activities /sessions for CG_2<br><br>Social chatting	For each group<br><br>-2 sessions/week for 6 weeks	Outcome measurement tools: Chinese version MMSE<br><br>Outcome evaluation: The Music-with-movement intervention improved global cognitive function with a medium effect size (Cohen's *d* = 0.51).
(Coelho et al., 2013)	EG = Exercise with cognitive training<br><br>CG = Treatment as usual	Activities /session for EG<br><br>- 60 min/session:<br><br>-5 min warm-up<br><br>-20 min aerobic	For EG<br><br>- 3 sessions/week for 16 weeks	Outcome measurement tools: Clock Drawing Test (CDT) & Frontal Assessment Battery (FAB) were used to measure executive function.<br><br>Outcome evaluation: Multimodal physical exercise positively affected frontal cognitive function with FAB (MD= 4.70) and CDT (MD = 1.0) among the intervention

		-35 min dual tasks which includes cognitive and motor training, i.e., walking, bouncing, language, counting backwards, generating words, weight training<br><br>-the complexity of the tasks increased every four weeks<br><br><u>Activities /sessions for CG</u><br><br>Treatment as usual but not involved in any of the exposed group activities		group, but patients in the control group worsened significantly in frontal cognitive function. The effect size was not reported in both assessment tools.
(de Andrade et al., 2013)	EG = Exercise with cognitive training<br><br>CG = Treatment as usual	<u>Activities /sessions for EG</u><br><br>- 60 min/session:<br><br>-5 min warm-up<br><br>-20 min aerobic<br><br>-35 min dual tasks, which include cognitive and motor training, i.e., walking, bouncing, language, count backwards, weight training<br><br>-the complexity of the tasks increased every four weeks	<u>For EG</u><br><br>- 3 sessions/week for 16 weeks	Outcome measurement tools: Montreal Cognitive Assessment (MoCA)<br><br>Outcome evaluation: The intervention improved global cognitive function among the intervention group compared with the control group; ($F(1,25) = 28.64$, $P < 0.001$), ES = 2.02 Cohen's *d*.

		<u>Activities /sessions for CG</u><br><br>Treatment as usual but involved in any of the exposed group activities		
(Graessel et al., 2011)	EG = Highly standardized intervention consisted of motor stimulation, the practice of activities of daily activity and cognitive stimulation (MAKS)<br><br>CG = Treatment as usual	<u>Activities /sessions for EG</u><br><br>-2 hours/session:<br><br>10 min round of greetings and group song (Spirituality)<br><br>-30 min motor exercises, such as bowling, croquet<br><br>- 10-minute break<br><br>- 30 minutes completing a variety of cognitive tasks like puzzling and picture drawing<br><br>-40min ADLs like preparing a snack<br><br><u>Activities /sessions for CG</u><br><br>Treatment as usual in residential care units	<u>For EG</u><br><br>-6 sessions/week for 48 weeks	Outcome measurement tools: Alzheimer's Disease Assessment Score – Cognitive subscale (ADAS-Cog)<br><br>*Note:* In this study, we consider per protocol analysis since they had a high number of dropouts (35/96).<br><br>Outcome evaluation: The intervention improved global cognitive function among the intervention group with the control group (ES = 0.45 Cohen's *d*)

(Higuti et al., 2020)	EG = Training with music (TWM)<br><br>CG = Training without music	<u>Activities /sessions for EG</u><br><br>-50 minutes/session:<br><br>-20 min songs of their time related to positive memories to stimulate cognition<br><br>-30 min set of light exercises for improving global mobility include rotational shoulder movements, hand flexion / extension, elbow pronation / supination, elbow flexion / extension etc.<br><br><u>Activities /sessions for CG</u><br><br>-30 min set of light exercises for improving global mobility include rotational shoulder movements, hand flexion / extension, elbow pronation / supination, elbow flexion / extension etc.	<u>For each group</u><br><br>-1 session/week for 12 weeks	Outcome measurement tools: MMSE<br><br>Outcome evaluation: There was no improvement or worsening cognitive function among either of the group but the effect size was not reported.
(Kampragkou et al., 2017)	EG = Combined aerobic, music therapy and memory game	<u>Activities /sessions for EG</u><br><br>-40 minute/session:	<u>For each group</u><br><br>-3 sessions/weeks for 12 weeks	Outcome measurement tools: MMSE

	CG = Only memory game	-30 minutes of aerobic exercise like walking<br><br>-10 minutes of memory games, attention and music therapy<br><br><u>Activities /sessions for CG</u><br><br>Memory game		Outcome evaluation: The intervention improved global cognitive function among the intervention group (ES = 0.96 Cohen's *d*).
(Kang et al., 2010)	EG = Integrated intervention of cognitive stimulation training, exercise, music, art, and horticultural therapy<br><br>CG = Treatment as usual	<u>Activities /sessions for EG</u><br><br>-3 hours/ sessions:<br><br>-5-min greeting<br><br>-10 min of warm-up hand exercises<br><br>-30-min cognitive stimulation activity<br><br>-30-min of music therapy<br><br>-10-min snack<br><br>-30 min of art therapy<br><br>-30 min of a horticulture activity	<u>For EG</u><br><br>-2 sessions/week for 9 weeks	Outcome measurement tool: Korean version MMSE<br><br>The median cognitive function was considered to evaluate the effect of intervention.<br><br>Outcome evaluation: The median cognitive measurement in intervention group is increased from 17.78 to 22.03 after treatment in the control group cognitive function decreased from 21.42 to 16.69.

		-30-minute second cognitive stimulation<br><br>-5-minute completion phase<br><br><u>Activities /sessions for CG</u><br><br>Treatment as usual		
(Karssemeijer et al., 2019)	EG = Exergame cognitive – aerobic bicycle training<br><br>CG_1 = Aerobic exercise<br><br>CG_2 = Active control	<u>Activities /sessions for EG</u><br><br>-40 minute/session:<br><br>Cycling stationary bicycle and simultaneously performed different cognitive tasks which was connected with video screen<br><br><u>Activities /sessions for CG_1</u><br><br>Cycling stationary bicycle was not connected to video screen for 40 minutes<br><br><u>Activities /sessions for CG_2</u><br><br>Relaxation and flexibility exercise for 30 minutes	<u>For each group</u><br><br>-3 sessions/week for 12 weeks	Outcome measurement tools: Specific cognitive functions like episodic memory, working memory, and psychomotor speed assessment was measured.<br><br>Outcome evaluation: There was moderate effect of exergame training and aerobic activity on only in psychomotor speed (MD = 0.283).
(Kim et al., 2016)	EG = Kohzuki Exercise Program (KEP) and Multi-component	<u>Activities /sessions for EG</u><br><br>One hour/session: KEP	<u>For EG</u><br><br>-5 sessions/week for 24 weeks	Outcome measurement tools: ADAS-cog

	Cognitive Program (MCP)<br><br>CG = Multi-component Cognitive Program (MCP)	-15 min of warm-up and stretching<br><br>-30 min of lower-limb aerobic exercise<br><br>-15 min of cool-down and relaxation<br><br>PLUS<br><br>One hour/session: MCP<br><br>-music therapy, art therapy, horticulture therapy, handicraft, recreational therapy and stretching<br><br><u>Activities /sessions for CG</u><br><br>One hour/session: MCP<br><br>-music therapy, art therapy, horticulture therapy, handicraft, recreational therapy and stretching	<u>For CG</u><br><br>-10 sessions/week for 24 weeks	Outcome evaluation:<br><br>ADAS-Cog score was significantly lower after 6 months in KEP plus MCP group than in the MCP group (F = 5.20, P = 0.03); ES = 0.29 Cohen's *d*.
(Lee et al., 2015)	EG = Combined fine motor skill and cognitive training<br><br>CG = general medical care	<u>Activities /sessions for EG</u><br><br>-60 minutes/session:<br><br>-Combined fine motor skill and cognitive training group consisted of coloring, singing, matching picture	<u>For EG</u><br><br>-3 sessions/week for 12 weeks	Outcome measurement tools: Korean version MMSE<br><br>Outcome evaluation: The intervention improved global cognitive function among the intervention group ($P <$ 0.05); ES = 1.52 Cohen's *d*.

		cards, playing instruments, physically interactive games, doing puzzles, chopsticks games, janggi football, and a fishing game<br><br><u>Activities /sessions for CG</u><br><br>General medical care only		
(Prick et al., 2017)	EG = Multicomponent dyadic intervention<br><br>CG = Minimum intervention in addition to the usual care	<u>Activities /sessions for EG</u><br><br>-one-hour long session/week for the first four weeks and 1-hr long session biweekly for second eight weeks multicomponent dyadic intervention<br><br>-30 minutes for physical exercise i.e., Strength, balance, flexibility and endurance exercises were included.<br><br>-10-15 min strength exercise<br><br>-10-15 min balance exercise	<u>For EG</u><br><br>-3 sessions/week for 12 weeks	Outcome measurement tools: Specific cognitive functions like memory, executive function and attention were measured independently.<br><br>A. Memory assessment was done by 8-word test and Rivermead Behavioral Memory Test (RBMT)<br>B. Executive function assessment was done by Behavioral Assessment of the Dysexecutive Syndrome (BADS), Groningen Intelligence Test (GIT) fluency animals and professionals<br>C. Attention assessment was done by Wechsler Memory Scale – Revised (WMS-R)<br>Outcome evaluation: There was a significant effect of the multicomponent dyadic intervention on the attention of people with dementia measured with the digit span forwards. No significant effects were found on the memory and executive function domains.

		-5-10 min flexibility with cooldown<br><br>- maximum of 20 min endurance/walking exercise<br><br>- psychoeducation, communication skill and pleasant activities<br><br>Activities / session for CG<br><br>There was minimal intervention in addition to the usual care like distributing bulletin monthly about dementia		
(Sampaio et al., 2019)	EG = Multicomponent Training (MT)<br><br>CG = Normal routine activities	Activities /sessions for EG<br><br>-50Min/session:<br><br>-5min Warm-up (including postural and stretching<br><br>-40 min moderate aerobic exercises<br><br>-5 min cooldown exercise (respiratory and flexibility exercises<br><br>Activities /sessions for CG	For EG<br><br>- 2 sessions/week for 24 weeks	Outcome measurement tool: MMSE<br><br>Outcome evaluation: Multicomponent group improved cognitive function in institutionalized older adults with mild to moderate dementia after six months of intervention with (ES = 0.88 Cohen's *d*).

		Normal routine activities		
(Satoh et al., 2017)	EG = exercise with music<br><br>CG = cognitive simulations	<u>Activities /sessions for EG</u><br><br>-40min/session:<br><br>The exercise consisted of muscle training for the upper and lower extremities, hand-clapping to music, breath and voice training, and singing.<br><br><u>Activities /sessions for CG</u><br><br>Cognitive activities like mistake searching in picture, maze, easy calculation	<u>For each group</u><br><br>- 1 session/week for 24 weeks	Outcome measurement tools: MMSE<br><br>Outcome evaluation: There was no improvement of global cognitive function
(Viola et al., 2011)	EG = Multidisciplinary rehabilitation program<br><br>CG = Waiting list for next intervention	<u>Activities /sessions for EG</u><br><br>-5hours/session:<br><br>-90 min Cognitive rehabilitation (computer-assisted)<br><br>-90 min Cognitive rehabilitation<br><br>-90 min Art therapy<br><br>-90 min Physical training	<u>For EG</u><br><br>-2 sessions/week for 12 weeks	Outcome measurement tools: MMSE<br><br>Outcome evaluation:<br><br>Outcome evaluation: There was no improvement of global cognitive function.

		-60 min Occupational therapy<br><br>-60 min Physiotherapy<br><br>-60 min Logic games like simple chess<br><br>-60 min Speech therapy<br><br>NB: 90 min for rest and lunch was provided<br><br><u>Activities / session for EG</u><br><br>Waiting list for next intervention		
(Young, 2020)	EG = Cognitive Stimulation Therapy (CST) and tai chi exercise<br><br>CG = Treatment as usual for elder in care centres	<u>Activities /sessions for EG</u><br><br>-one hour cognition activities of reality orientation, physical games, food, sounds, childhood, senses, faces, number games, word games, current events, categorising objects, and tai chi exercise<br><br><u>Activities /sessions for CG</u><br><br>Treatment as usual for elder in care centres but not	<u>For each group</u><br><br>-2 sessions /week for 7 weeks	Outcome measurement tools: MMSE<br><br>Outcome evaluation: The combined intervention significantly improved MMSE (with moderate to large effect size (F = 9.96,); ES = 0.70 Cohen's *d*.

		cognitive training and tai chi exercise		
(Young et al., 2019)	EG = Cognitive Stimulation Therapy (CST) and tai chi exercise<br><br>CG = Treatment as usual for elder in care centres	<u>Activities /sessions for EG</u><br><br>-one hour cognition activities of reality orientation, physical games, food, sounds, childhood, senses, faces, number games, word games, current events, categorising objects, and tai chi exercise<br><br><u>Activities /sessions for CG</u><br><br>Treatment as usual for elder in care centers but not cognitive training and tai chi exercise	<u>For each group</u><br><br>-2 sessions /week for 7 weeks	Outcome measurement tools: MMSE<br><br>Outcome evaluation: The combined intervention significantly improved MMSE (F = 12.31); ES = 0.71 Cohen's *d*.

Abbreviations: EG: Exposed group; CG: Control group; ES: Effect size; MD: Mean difference
