# Supplementary material for: The Effect of Multimodal Non-pharmacological Interventions on Cognitive Function Improvement for People With Dementia: A Systematic Review
Source: Front Public Health. 2022 Jul 12;10:894930. doi: 10.3389/fpubh.2022.894930 (PMC9314571; doi:10.3389/fpubh.2022.894930)
Supplement: Supplementary file 3 [file Table_3.pdf]

Supplementary Table 3: Search string for each database

S.no	Name of the database	Search string
1.	PubMed	(("Dementia"[Mesh] OR Dementi*[tiab] OR Alzheimer*[tiab]) AND (Combin*[tiab] OR multimodal[tiab] OR multidomain[tiab] OR Multicomponent[tiab] OR Mixed[tiab] OR Integrated[tiab]) AND ("Aged"[Mesh] OR "Homes for the Aged"[Mesh] OR elderly[tiab] OR senior*[tiab] OR "older patient*"[tiab] OR "older people"[tiab] OR "older person*"[tiab] OR "older ag*"[tiab] OR "old age"[tiab] OR "older adult*"[tiab] OR "older residents"[tiab] OR geriatr*[tiab]) AND ("Cognition"[Mesh] OR memory [tiab] OR "Executive Function"[Mesh] OR cogniti*[tiab] OR "Cognitive function*"[tiab] OR "Executive function*"[tiab]) AND ("Randomized Controlled Trial" [Publication Type] OR "Randomized controlled trial*"[tiab]))
2.	Embase	('dementia'/exp OR 'dementia' OR dementi*:ab,ti OR alzheimer*:ab,ti) AND (combin*:ab,ti OR multimodal:ab,ti OR multidomain:ab,ti OR multicomponent:ab,ti OR mixed:ab,ti OR integrated:ab,ti) AND ('aged'/exp OR 'aged' OR 'senior center'/exp OR 'senior center' OR 'home for the aged'/exp OR 'home for the aged' OR elderly:ab,ti OR senior*:ab,ti OR 'older patient*':ab,ti OR 'older people':ab,ti OR 'older ag*':ab,ti OR 'old age':ab,ti OR 'older adult*':ab,ti OR 'older residents':ab,ti OR geriatr*:ab,ti) AND ('cognition'/exp OR 'cognition' OR 'executive function'/exp OR 'executive function' OR (((memory:ab,ti OR cogniti*:ab,ti OR cognitive:ab,ti) AND function*:ab,ti OR executive:ab,ti) AND function*:ab,ti)) AND ('randomized controlled trial'/exp OR 'randomized controlled trial' OR 'randomized controlled trial*':ab,ti)
3.	CINAHL	(((MH "Dementia+") OR TI (Dementi* OR Alzheimer*) OR AB (Demnti* OR Alzheimer*))) AND ((TI (Combin* OR multimodal OR multidomain OR Multi Component OR Mixed OR Integrated) OR AB (Combin* OR multimodal OR multidomain OR Multi Component OR Mixed OR Integrated))) AND ((MH "Aged+" OR MH "Aged, Hospitalized" OR TI elderly OR AB elderly OR TI senior* OR AB senior* OR TI "older patient*" OR AB "older patient*" OR TI "older people" OR AB "older people" OR TI "older ag*" OR AB "older ag*" OR TI "old age" OR AB "old age" OR TI "older adult*" OR AB "older adult*" OR TI "older residents" OR AB "older residents" OR TI geriatr* OR AB geriatr*)) AND ((MH "Cognition+") OR (MH "Executive Function") OR AB (Memory OR Cogniti* OR “Cognitive function*” OR “Executive function*”) OR TI (Memory OR Cogniti* OR “Cognitive function*” OR “Executive function*”)) AND (((MH "Randomized Controlled Trials+") OR TI “Randomized controlled trial*”) OR AB (“Randomized controlled trial*”)))
4.	Medline	(((MH "Dementia+") OR TI (Dementi* OR Alzheimer*) OR AB (Demnti* OR Alzheimer*))) AND ((TI (Combin* OR multimodal OR multidomain OR Multi Component OR Mixed OR Integrated) OR AB (Combin* OR multimodal OR multidomain OR Multi Component OR Mixed OR Integrated))) AND ((MH "Aged+" OR MH "Aged, Hospitalized" OR TI elderly OR AB elderly OR TI senior* OR AB senior* OR TI "older patient*" OR AB "older patient*" OR TI "older people" OR AB "older people" OR TI "older ag*" OR AB "older ag*" OR TI "old age" OR AB "old age" OR TI "older adult*" OR AB "older adult*" OR TI "older residents" OR AB "older residents" OR TI geriatr* OR AB geriatr*)) AND ((MH "Cognition+") OR (MH "Executive Function") OR AB (Memory OR Cogniti* OR “Cognitive function*” OR “Executive function*”) OR TI (Memory OR Cogniti* OR “Cognitive function*” OR “Executive function*”)) AND (((MH "Randomized Controlled Trials+") OR TI “Randomized controlled trial*”) OR AB (“Randomized controlled trial*”)))

5.	Web of science	TS= (Dementia OR Dementi* OR Alzheimer*) AND TS= (Combin*OR multimodal OR multidomain OR Multicomponent OR Mixed OR Integrated) AND TS= (Aged OR "Homes for the Aged" OR elderly OR senior*OR "older patient*" OR "older people" OR "older person*"OR "older ag*" OR "old age" OR "older adult*" OR "older residents" OR geriatr*) AND TS= (Cognition OR memory OR "Executive Function" OR cogniti* OR "Cognitive function*" OR "Executive function*") AND TS= ("Randomized Controlled Trial" OR "Randomized controlled trial*)
