# Supplementary figures and images for: The Effect of Multimodal Non-pharmacological Interventions on Cognitive Function Improvement for People With Dementia: A Systematic Review
Source: Front Public Health. 2022 Jul 12;10:894930. doi: 10.3389/fpubh.2022.894930 (PMC9314571; doi:10.3389/fpubh.2022.894930)

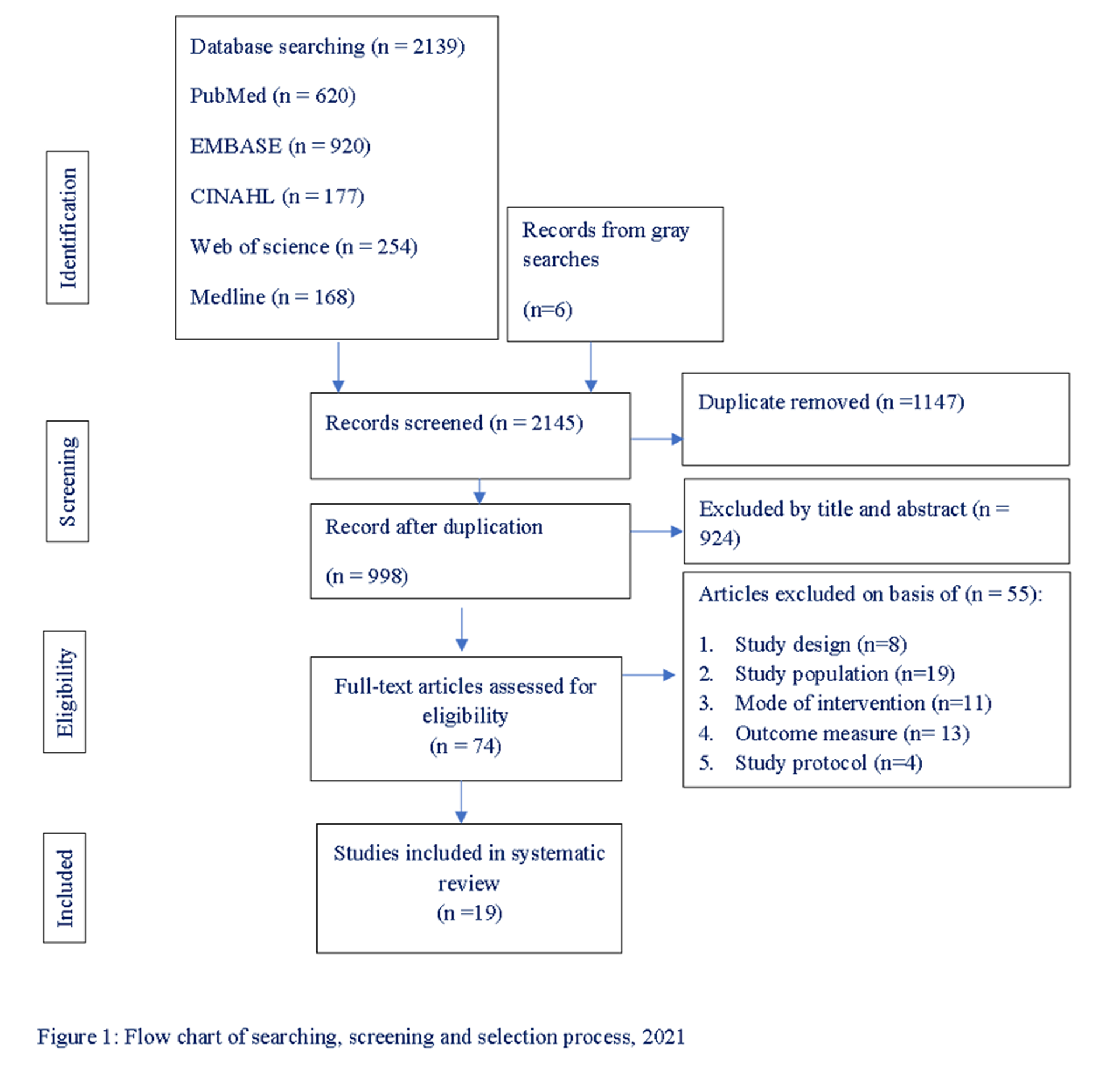

Supplement: Supplementary file 4 [file Image_1.png]

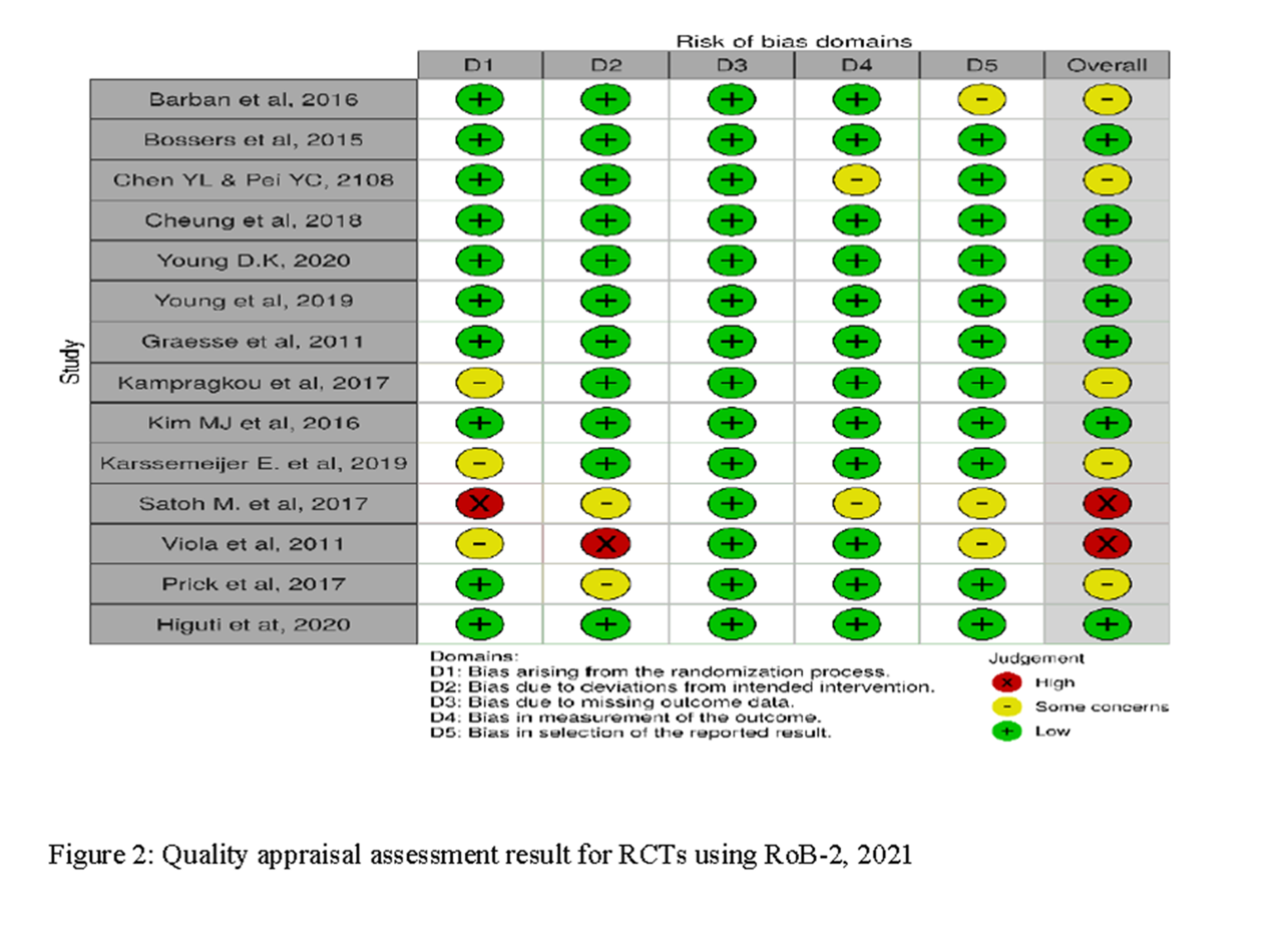

Supplement: Supplementary file 5 [file Image_2.png]

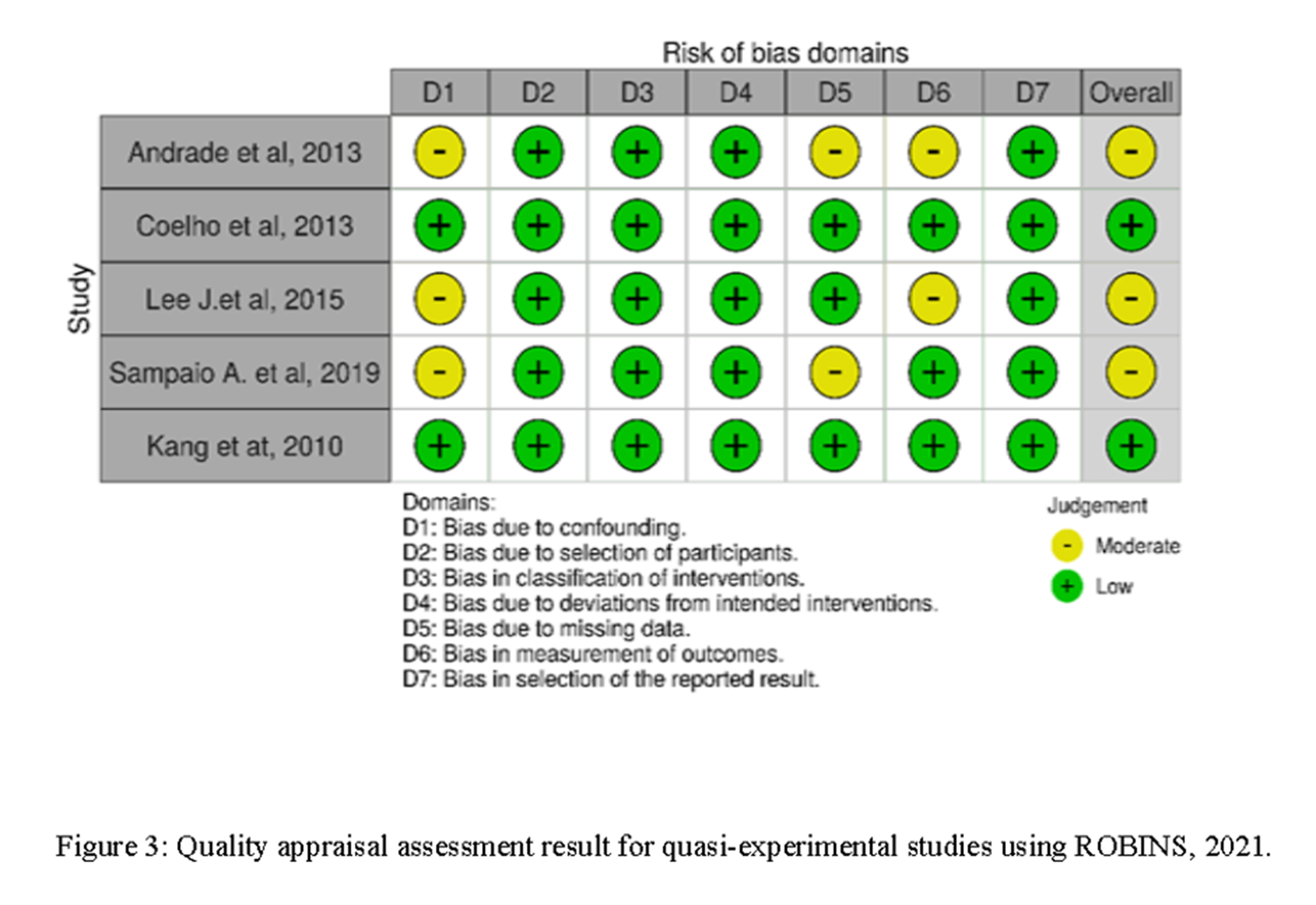

Supplement: Supplementary file 6 [file Image_3.png]
